# Mesenchymal Stem Cells and Their Extracellular Vesicles Are a Promising Alternative to Antibiotics for Treating Sepsis

**DOI:** 10.3390/bioengineering11111160

**Published:** 2024-11-18

**Authors:** Yu Jiang, Yunjuan Song, Qin Zeng, Bin Jiang

**Affiliations:** 1 Key Laboratory of Birth Defects and Related Diseases of Women and Children, Sichuan University, Chengdu 610041, China; 2 R&D Division, Eureka Biotech Inc., Philadelphia, PA 19104, USA; 3 National Engineering Research Center for Biomaterials, Sichuan University, Chengdu 610065, China

**Keywords:** sepsis, antibiotics resistance, mesenchymal stem cells, immune-modulatory, bioengineering

## Abstract

Sepsis is a life-threatening disease caused by the overwhelming response to pathogen infections. Currently, treatment options for sepsis are limited to broad-spectrum antibiotics and supportive care. However, the growing resistance of pathogens to common antibiotics complicates treatment efforts. Excessive immune response (i.e., cytokine storm) can persist even after the infection is cleared. This overactive inflammatory response can severely damage multiple organ systems. Given these challenges, managing the excessive immune response is critical in controlling sepsis progression. Therefore, Mesenchymal stem cells (MSCs), with their immunomodulatory and antibacterial properties, have emerged as a promising option for adjunctive therapy in treating sepsis. Moreover, MSCs exhibit a favorable safety profile, as they are eventually eliminated by the host’s immune system within several months post-administration, resulting in minimal side effects and have not been linked to common antibiotic therapy drawbacks (i.e., antibiotic resistance). This review explores the potential of MSCs as a personalized therapy for sepsis treatment, clarifying their mechanisms of action and providing up-to-date technological advancements to enhance their protective efficacy for patients suffering from sepsis and its consequences.

## 1. Introduction

Sepsis is a systemic inflammatory response syndrome caused by various pathogens, including bacterial, viral, fungal, and parasitic infections. The early symptoms of sepsis are often subtle and can easily be confused with other illnesses, leading to delayed treatment.

In clinical practice, sepsis is often treated with antibiotics to control the infection quickly. The principles of antibiotic use are crucial, including early use, targeted use, combined use, and adequate dosage and duration of treatment [1,2]. These principles ensure that antibiotics can kill pathogenic bacteria as quickly as possible, prevent the spread of infection, and gain valuable treatment time for patients. However, the long-term or improper use of antibiotics has led to a severe issue: the gradual development of bacterial drug resistance under the screen of antibiotics [3,4]. Gradually, these drug-resistant bacteria become dominant and can withstand or evade the killing effect of antibiotics, persisting and multiplying in patients. This poses a significant and ongoing challenge to the treatment of sepsis. For example, there is a rising global incidence of neonatal sepsis caused by extended-spectrum beta-lactamase (ESBL), which produces multi-drug resistant (MDR) Escherichia coli (*E. coli*). Zhu et al. reported that ESBL-producing MDR *E. coli* sepsis is more prevalent in premature infants and those with hospital-acquired late-onset sepsis (HALOS). The mortality rate for neonatal sepsis caused by ESBL-producing *E. coli* is approximately twice that of sepsis caused by ESBL-negative *E. coli* [3]. Therefore, the successive development of bacterial drug resistance affects the therapeutic effect of sepsis and may lead to more severe infections.

**Figure 1:**
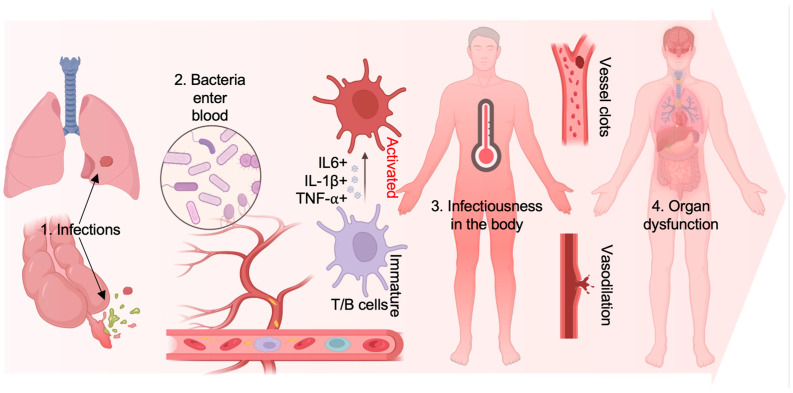
Illustration of the Progression Stages in Sepsis Development. The initial phase, Systemic Inflammatory Response Syndrome (SIRS), can originate from infections in organs such as the lungs and appendix. Subsequently, the dissemination of bacteria into the bloodstream triggers inflammation, characterized by the activation of T and B cells and the elevation of pro-inflammatory cytokines, including IL-6, IL-1β, and TNF-α. This process is also associated with an increase in body temperature. As the infection advances, vascular thrombosis occurs, marking the transition to the second stage, severe sepsis. The onset of acute organ dysfunction distinguishes this stage. It can be identified in sepsis with accompanying hypotension (low blood pressure) or hypoperfusion (reduced blood flow through an organ). Without effective intervention, the condition may progress to the third stage, septic shock, characterized by a bacterial infection leading to low blood pressure, vasodilation, and organ failure.

On the other hand, sepsis presents with a range of clinical symptoms, including those associated with systemic inflammatory response syndrome (SIRS), abnormal body temperature, increased heart rate, rapid breathing, blood circulation disorders (e.g., hypotension and shock), and metabolic disorders [5]. Treatment typically involves using antibiotics, vasoactive drugs, and fluid resuscitation to alleviate these symptoms. However, one of the most challenging aspects of sepsis is the overreaction of the body’s immune system due to individual variations. This overreaction produces many inflammatory factors (cytokines), resulting in a cytokine storm [6,7,8]. Specifically, it happens when an infectious agent-like bacteria enters the bloodstream, immune cells such as macrophages and T cells are activated and release numerous cytokines, including tumor necrosis factor (TNF-α), interleukins (IL-1, IL-6), and interferons (IFN-γ), against infection [9,10]. However, their excessive release during sepsis can cause an uncontrolled inflammatory response and lead to extensive tissue damage and organ dysfunction, posing a serious threat to life (Figure 1), which is like what happened with COVID-19. Therefore, effectively controlling the cytokine storm caused by inflammation is crucial in treating sepsis.

Amidst these challenges, recent studies have revealed that mesenchymal stem cells (MSCs) possessed antibacterial properties and excellent immune-modulatory [11,12,13,14]. They can secrete antibacterial molecules that directly interact with pathogens and factors that enhance the antibacterial activity of host immune cells. They also modulate the hyperactive immune response by secreting anti-inflammatory factors like interleukin-1 receptor antagonist (IL-1Ra) and interleukin-10 (IL-10), activating the T regulatory cells (T_reg_ cells) and protecting organs from septic shock. These unique properties position MSCs as potential alternatives to antibacterial drugs, offering a glimmer of hope in the battle against bacterial drug resistance. Studies on human umbilical cord mesenchymal stem cells (UC-MSCs) for treating pneumonia-induced sepsis have shown positive results in several trials, bolstering confidence in the safety and efficacy of this approach [11,15]. In this review, we collect the latest research on the combination of MSC therapy. Moreover, we elaborate on its safety profile to understand the pathophysiology of sepsis and explore promising advancements in managing this critical condition.

## 2. The Administration of Sepsis and Emerged Antibiotic Resistance

### 2.1. Generation of Antibiotic Resistance in Microbes During Sepsis

Sepsis is a serious disease caused by pathogenic microorganisms invading the blood circulation system and causing systemic infection. Antibiotics can effectively inhibit or kill these pathogenic microorganisms, thereby controlling the infection and alleviating the condition [16,17].

In the natural environment, resistance can arise through genetic mutations or the acquisition of resistance genes from other bacteria in a slow process [18,19]. These genes can enable bacteria to neutralize antibiotics, alter drug targets, or prevent drug entry into the cell. In contrast, antibiotic treatment for sepsis can disrupt the normal microbiota, allowing resistant strains to dominate much faster. For instance, Pyelonephritis, a type of urinary tract infection (UTI) that affects the kidneys, can be particularly severe when caused by resistant strains of Escherichia coli (*E. coli*). Two notable types of resistant *E. coli* are those that produce extended-spectrum β-lactamases (ESBLs) and those resistant to fluoroquinolone treatment [20]. Vancomycin-resistant Enterococcus (VRE) can dominate the intestinal microbiota following antibiotic treatment, increasing the risk of bloodstream infections [21]. In clinical cases, overuse and misuse of antibiotics cause antibiotic resistance as accelerated bacteria change to resist antibiotics used to treat them effectively. This makes certain bacterial infections in sepsis extremely difficult to treat [22].

Under antibiotic pressure, bacteria spontaneously adapt to the environment through genetic mutations. The spread of drug-resistant genes became an essential factor leading to the development of bacterial resistance. Resistance genes spread among bacteria through horizontal transfer and other methods, causing bacteria that were initially sensitive to antibiotics to become resistant and make bacterial infections very difficult to control.

### 2.2. The Conventional Treatments for Sepsis

The sepsis pathophysiology involves immune dysregulation and coagulopathy, which can present multiple symptoms depending on the individual conditions. The main strategies for sepsis treatment focus on cholinergic anti-inflammatory pathways and extracorporeal techniques to modulate the immune response and protect against sepsis-induced consequences [23,24,25].

Early recognition and timely initiation of treatment are essential to maintaining the health of sepsis patients. Antibiotic therapy is the primary treatment as soon as sepsis is noticed. Firstly, broad-spectrum antibiotics are used initially to cover possible pathogens. Once the pathogen is identified based on the results of blood cultures., antibiotic therapy may be adjusted accordingly throughout the whole process of disease [26]. Meanwhile, intravenous fluid resuscitation is administered to maintain blood pressure and ensure enough blood flow to organs. Commonly used fluids include normal saline or lactated Ringer’s solution [27]. Fluid therapy is critical in the early stage of sepsis onset to prevent septic shock.

In addition to antibiotic therapy and fluid resuscitation, vasopressors are needed when fluid resuscitation is insufficient to maintain normal blood pressure. When infection expands to the whole body, it is important to ensure that vital organs receive enough nutrients and oxygen in the blood [28]. If sepsis develops to a late stage and organ dysfunction happens, such as respiratory failure and kidney failure, mechanical ventilation and dialysis are needed, respectively, as organ-supportive methods to support any functionally failing organs and prevent the death of patients [29].

Nevertheless, the ultimate goal of sepsis treatment is to prevent the progression of sepsis to septic shock and multiple organ dysfunction syndrome (MODS), which may significantly increase the risk of death and require the transfer of patients to intensive care units (ICUs) [30]. Although conventional therapies for sepsis have made significant progress, it is still a challenge to control the overactivated immune response per patient under different conditions. Therefore, research is ongoing into the use of immunomodulatory drugs, such as monoclonal antibodies [31,32] and MSCs [33,34], to regulate the inflammatory response, but these therapies are mainly in the experimental stages.

### 2.3. The Shortages of the Conventional Treatment for Sepsis

Treating sepsis involves a combination of therapies to fight the infection, support organ function from septic shock, and prevent further organ dysfunction. However, several challenges and shortages are associated with the conventional treatment of sepsis when facing the losing control of the overactivated immune response (i.e., cytokine storm) above mentioned, and particularly the concern of antibiotic resistance, which is one of the curbs of sepsis treatment, which involves the misuse of antibiotics to fight the underlying infection [35,36]. Moreover, the increasing prevalence of antibiotic-resistant bacteria may pose a significant challenge, making it more difficult to treat future infections effectively and necessitating the incremental use of more potent, often more cytotoxic, and expensive antibiotics [37].

Many researchers focus on understanding the mechanisms and implications of antibiotic resistance in treating sepsis to combat this growing threat. Iredell et al. delve into the mechanisms and clinical consequences of antibiotic resistance in Enterobacteriaceae, shedding light on the mobility and fixation of resistance genes [38]. Meanwhile, Atif et al. highlight the importance of rational antibiotic use in reducing adverse outcomes in neonatal sepsis patients, emphasizing the need for tailored treatment approaches [39]. On the correlation between antibiotic resistance and sepsis incidence in community-acquired bacterial pneumonia, Curren et al. focus on advancing diagnostic stewardship for healthcare-associated infections, antibiotic resistance, and sepsis, emphasizing the importance of improved outcomes and patient safety through strategic interventions [40].

On the other hand, there are other limitations of sepsis treatments on early detection and diagnosis challenges. Early detection and diagnosis of sepsis are critical for effective management, but symptoms can be nonspecific and varied in practice, leading to poor outcomes [41,42]. Although there are emerging novel technologies based on biomarkers associated with innate response, such as pathogen-associated molecular patterns (PAMPs) and damage-associated molecular patterns (DAMPs), designed to help identify sepsis at an early stage shown in Table 1; however, there are limitations either by cost or reliability in practical diagnostic tests for sepsis, complicating early and precise identification [43]. Moreover, the limitations of sepsis treatments on fluid management and vasopressor use are not ignorable because managing fluids and using vasopressors to support blood pressure are the critical aspects of sepsis treatment at the early stage [28,44]. Those uncertainties may lead to variability in treatment approaches and outcomes.

## 3. Mesenchymal Stem Cells Serve as a Novel Avenue to Attenuate Sepsis

### 3.1. Origins and Characters of Mesenchymal Stem Cells

**Figure 2:**
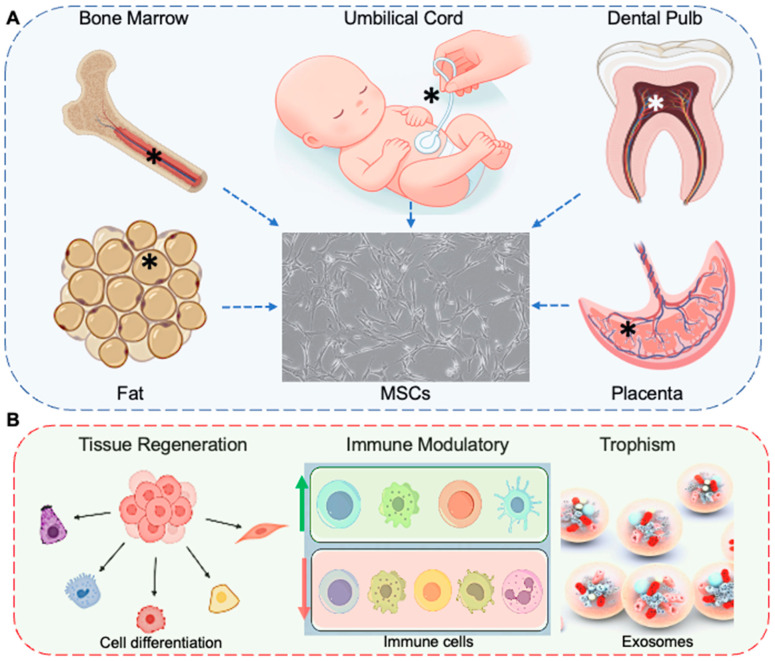
Overview of MSCs origins and their therapeutic role in sepsis. (**A**) MSCs are sourced from various bodily tissues, including bone marrow, adipose tissue, umbilical cord and its blood, dental pulp, and placenta. These cells adhere to 2D culture surfaces, exhibiting a fibroblast-like cell morphology. MSCs can undergo several rounds of expansion; however, only cells from early passages (e.g., up to 6 passages) are considered suitable for therapeutic applications. (**B**) MSCs can differentiate into various somatic cell types, such as muscle cells, osteocytes, chondrocytes, and adipocytes, contributing to tissue regeneration. MSCs exhibit immunomodulatory effects by inhibiting Th1/17 cells, M1 macrophages, natural killer cells, plasma cells, and neutrophils while promoting the activity of regulatory T cells, M2 macrophages, and dendritic cells. This modulation of the immune response, combined with the secretion of exosomes containing beneficial molecules, peptides, and microRNAs, enables MSCs to offer organ protection and prevent the progression of septic shock. * Indicates the donor samples.

MSCs are multipotent stromal cells originating from the mesoderm that may derive from multiple somatic tissues, such as bone marrow, adipose tissue, umbilical cord blood, and dental pulp, as shown in Figure 2A. Bone marrow and fat tissue are the most common sources of MSCs used for clinical research [53]. MSCs are normal fibroblast-like cells in 2D culture in vitro; when dissociated to be single cells, they need to be characterized by specific surface biomarkers, including CD105, CD73, and CD90, while lacking hematopoietic biomarkers, including CD34, CD45, CD11b, or CD14, CD79α or CD19, and HLA-DR, to match the minimum requirement issued by the International Society for Stem Cells Research (ISSCR) [54,55].

In addition, one significant profile of MSCs is their low immunogenicity, which facilitates their application in allogeneic transplantations, allowing for the development of off-the-shelf therapies that can be administered without the need for donor–recipient matching [56]. This broadens the potential application of MSC-based treatments, making them a versatile and valuable tool in regenerative medicine, and has already shown positive outcomes for human clinical trials (Table 2).

### 3.2. MSCs Exert Multiple Bioactivities for Sepsis Administration

As shown in Figure 2B, MSCs play a pivotal role in regenerative medicine and tissue repair thanks to their unique functions that extend beyond their ability to differentiate into various cell types [78]. One of the hallmark functions of MSCs is their capacity for self-renewal and differentiation, which enables them to contribute to the regeneration and repair of mesenchymal tissues such as bone, cartilage, fat, and muscle tissues [78]. This characteristic is fundamental to their application in addressing conditions like osteoarthritis, muscular dystrophies, and bone fractures, where the regeneration of tissue integrity is crucial [79].

Beyond tissue regeneration, MSCs exert significant immunomodulatory effects [80]. They can modulate the responses of various immune cells, including T cells, B cells, dendritic cells, and natural killer cells, thereby playing a critical role in reducing inflammation and promoting tissue healing [81]. This immunomodulatory function is particularly beneficial in treating autoimmune diseases, preventing organ transplant rejection, and managing graft-versus-host disease (GVHD), showcasing their potential as therapeutic agents in immune-mediated conditions [81].

MSCs also secrete many bioactive molecules, such as growth factors, cytokines, and chemokines, contributing to their trophic effects (usually via the exosomes) [82]. These secretions can enhance the repair of damaged tissues, promote angiogenesis (the formation of new blood vessels), and support the survival and function of other cells within the local microenvironment. This paracrine signaling mechanism is critical in MSC-mediated tissue repair and regeneration, highlighting their role as mediators of cellular communication and tissue homeostasis [83]. Together, these characters underscore the multifaceted role of MSCs in promoting healing, modulating immune responses, and restoring tissue function, making them a cornerstone of current and future therapeutic strategies [79].

### 3.3. Current Application of MSCs for Diseases

MSCs have emerged as a promising therapeutic tool for treating various diseases. This literature review aims to provide a comprehensive overview of the current application of MSCs for different diseases, drawing insights from recent research and clinical trials. For example, the potential use of MSCs in treating diabetes has been a subject of research interest. Gao et al. presented an overview of the current status of MSC therapy for Type 2 Diabetes (T2DM), emphasizing the mechanism of the pharmacal kinetics of transplanted cells and discussing the hurdles in MSC-based T2DM therapy [84]. Additionally, Suchanek and Xu et al. demonstrated the promotion of differentiation of human dental pulp mesenchymal stem cells into insulin-producing cells, suggesting a potential avenue for improving diabetes therapy [85,86].

In the context of cardiovascular diseases, the therapeutic potential of MSCs for ischemic heart disease has been a focus of research. Karantalis et al. and Singh et al. discussed using mesenchymal stem cells to improve cardiac function and vascular regeneration, particularly in ischemic heart disease [87,88]. Moreover, Bagno et al. reviewed the rational use of MSCs in treating ischemic heart disease [89], emphasizing the mechanisms of MSC-based cardiac regeneration and novel approaches for enhancement. Moreover, Hmadcha et al. provided an overview of the therapeutic potential of MSCs for cancer treatment, with a specific emphasis on central nervous system tumors [90]. Similarly, Wang et al. discussed the advances in MSC therapy for immune and inflammatory diseases, shedding light on the outcomes of current clinical trials and emerging trends in the field [12]. These papers collectively contribute to understanding the diverse therapeutic applications of MSCs in the context of cancer and immune-inflammatory conditions. Furthermore, the application of MSC-based therapy in modulating the tumor microenvironment, particularly in breast cancer, has been explored. Soufiani et al. investigated the potential of using a combination of microRNA-34a and MSC-conditioned medium to change the cancerous microenvironment and its implications for breast cancer therapy [91].

In conclusion, the literature on the application of MSCs for various diseases demonstrates the diverse therapeutic potential of these cells. Some MSC products have already been approved for commercialized drugs (Table 3). The findings from these studies collectively contribute to the advancement of MSC-based therapies and provide valuable insights for future research and clinical applications.

## 4. The Beneficial Characteristics of MSCs for Sepsis Treatment

### 4.1. Anti-Bacteria Effect of MSCs

Studies have shown that MSCs can combat bacterial infections and regulate the immune response in sepsis, making them a promising therapeutic option [92]. The antibacterial effect of MSCs, particularly against Escherichia coli, has been attributed to the secretion of beta-defensin-2 (BD2) via the toll-like receptor 4 (TLR-4) signaling pathway [92]. This indicates that MSCs can directly target pathogenic bacteria and enhance the host’s defense mechanism against infection. In addition, the potential application of MSCs in acute lung injury (ALI) has been highlighted, emphasizing the therapeutic mechanisms involving secreted paracrine soluble factors such as antimicrobial peptides (AMPs) [93]. This suggests that MSCs could potentially be utilized in treating sepsis-induced lung injury by leveraging their paracrine effects. Furthermore, the assessment of MSC therapy in acute and chronic liver failure and chronic liver disease has shown promising results in improving liver function, indicating the potential of MSCs to alleviate organ dysfunction associated with sepsis [94]. Additionally, MSCs have been demonstrated to exert anti-inflammatory and antibacterial effects in a mouse model of chronic obstructive pulmonary disease (COPD)-induced lung injury, further supporting their potential in combating sepsis-associated lung complications [95].

The regulatory role of MSCs in modulating macrophage polarization for treating sepsis-induced liver injury has been a subject of interest, providing insights into the immunomodulatory mechanisms of MSCs in sepsis [96]. Moreover, the effects of human umbilical cord-derived MSCs in protecting endothelial and tubular cells in sepsis-induced acute kidney injury have been investigated, shedding light on the potential protective mechanisms of MSCs in sepsis-related organ damage [97]. Apart from their direct antibacterial effects, MSCs have also been implicated in modulating protein phosphorylation mechanisms to prevent and intervene in the development of sepsis, which could offer a novel target for therapeutic intervention [15]. In addition, the regulatory effects of MSCs on macrophages in sepsis have been explored, providing valuable insights into the immunological mechanisms and clinical applications of MSCs in sepsis treatment [98].

While these studies suggest the potential of MSCs in treating sepsis with cytokines and AMPs, it is essential to note that the safety and efficacy of MSC therapy for sepsis treatment are being actively investigated [11]. A protocol study for a clinical trial assessing the safety and efficacy of human umbilical cord MSCs in treating sepsis induced by pneumonia highlights the ongoing efforts to advance the understanding of MSCs in sepsis treatment [99,100]. In conclusion, the literature on MSCs in sepsis treatment demonstrates their multifaceted potential, from their direct antibacterial effects to their immunomodulatory and organ-trophism properties [101,102]. The ongoing research and clinical trials in this field underscore the significance of further exploring the therapeutic application of MSCs for sepsis management.

### 4.2. Immunomodulatory Effect of MSCs for Sepsis

The immunomodulatory effects of MSCs have garnered significant attention as a potential treatment for sepsis. Understanding the mechanisms underlying the immune-regulating properties of MSCs is crucial for harnessing their therapeutic potential in sepsis [103]. Weiss and Dahlke comprehensively review the mechanisms of action of living, apoptotic, and dead MSCs in immunomodulation [103]. The viability of MSCs is a focal point in this review, addressing concerns about the tumorigenic potential of living MSCs. This review delves into the cellular and molecular mechanisms involved in MSC-mediated immunomodulation, shedding light on their potential for sepsis treatment [103].

Similarly, studies have demonstrated the potential of MSC-derived extracellular vesicles (MSC-EVs) as a novel therapeutic strategy for sepsis. Zheng et al. discuss the regenerative and immunomodulatory effects of MSC-EVs, highlighting their potential application in sepsis treatment. This emphasizes the multifaceted nature of MSCs and their components in modulating immune responses, thus offering promising avenues for sepsis therapy [104]. Wu et al. showcase the immunomodulatory effects of human umbilical cord-derived MSCs in alleviating acute lung injury (ALI) through the MyD88-NFκB signaling pathway [105]. This study underscores the therapeutic potential of MSCs in mitigating the cytokine storm associated with sepsis-induced ALI, thereby reinforcing the immunomodulatory role of MSCs in a clinical setting. In a related vein, Deng et al. reveal that exosomes derived from different sources of human MSCs effectively downregulate sepsis-induced glycolysis and inflammation, thus easing lung pathological damage [106]. Caplan et al. summarized studies demonstrating that MSCs were mobilized from their perivascular niches, subsequently activating to regenerate the microenvironment by secreting bioactive molecules and modulating the local immune response [107]. Furthermore, they emphasized that identifying distinct innate functional MSC subpopulations had the potential to enhance therapeutic efficacy [108].

Those findings underscore the diverse immunomodulatory effects of MSC-derived exosomes, further underlining the potential for these components in sepsis treatment.

### 4.3. Trophism of MSCs Prevents the Organs from Septic Shock

A critical aspect in the pathophysiology of sepsis and the genesis of septic shock is the interaction between MSCs and mediators of immunity and tissue repair. This interaction is essential to proposing paradigms related to the precise roles of MSCs in sepsis and septic shock [33]. Notably, it has been demonstrated that exosomes derived from different sources of MSCs effectively downregulated sepsis-induced glycolysis and inflammation, facilitated lung pathological damage, and improved the survival rate of mice with sepsis [109]. Furthermore, MSC treatment of preclinical sepsis has been shown to significantly reduce mortality over various experimental conditions, indicating a potential mortality rate reduction through MSC therapy [110,111]. Moreover, recent findings highlight the potential of MSCs and MSC-derived secretomes in improving sepsis-induced organ dysfunction, including reduced bacterial load and decreased levels of pro-inflammatory factors and further inhibiting apoptosis at the cellular level via trophic effect [100,112].

**Figure 3:**
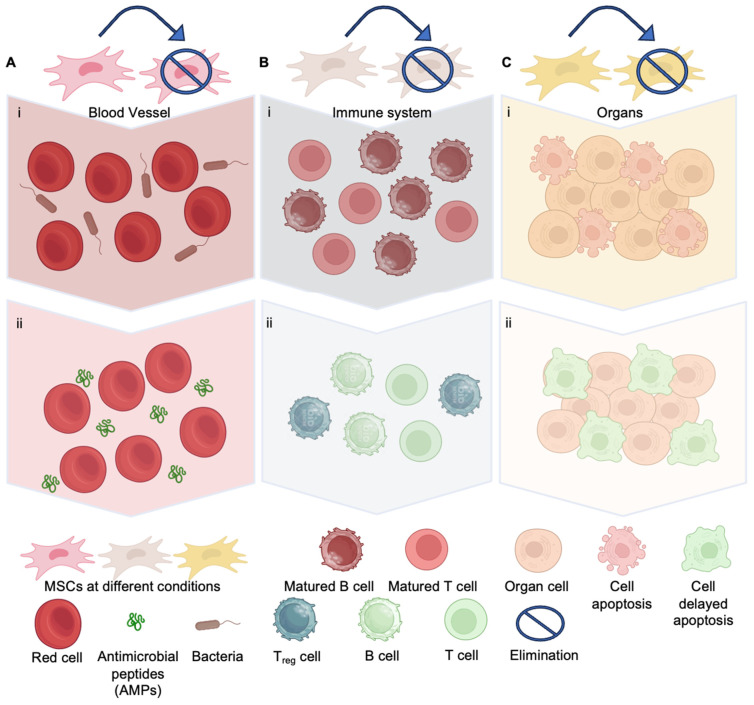
The role of MSCs in sepsis treatment: a promising therapeutic strategy. (**A**,**B**) MSCs offer a novel personalized approach to sepsis therapy, including autogenous or allogenous avenues, differing from traditional antibiotics, which solely target pathogens without addressing the inflammatory cascade or subsequent organ damage. MSCs have unique attributes that render them particularly efficacious in combating sepsis. They can produce antimicrobial peptides (AMPs) (**A**) and modulate immune responses (**B**), specifically by inhibiting the maturation of T and B cells and enhancing the proportion of regulatory T cells. This dual action facilitates infection control and diminishes the systemic inflammation that is a hallmark of sepsis. (**C**) Additionally, MSCs have an extraordinary ability to provide organ protection through trophic effects, halting the advancement to septic shock by maintaining organ function and fostering tissue repair and regeneration. Such trophic support significantly improves recovery chances for individuals experiencing septic shock. Eventually, all the MSCs are eliminated by the host immune system; the elapse from hours to weeks varies per the location in vivo, which promises a high safety profile. For the images labeled as “i” means before MSCs administration and “ii” means post-MSCs administration.

In a preclinical study, treatment with human umbilical cord-derived MSCs seems to protect endothelial and tubular cells in sepsis-induced acute kidney injury, possibly via the TLR4/NF-κB signaling pathway [97]. The immunomodulatory properties, antimicrobial activity, and protection capacity (i.e., trophic) against organ failure make MSCs a promising therapy for sepsis and septic shock [33,113]. The ability of MSCs to control the inflammatory response induced by sepsis by regulating Th cells and inflammatory factors has been shown to reduce tissue damage, protect organ functions, and improve survival in aged sepsis model rats [77]. These numerous studies shed light on the potential of MSCs in treating sepsis and septic shock, from their immunomodulatory effects to their therapeutic impact on different affected organs. MSCs offer a multifaceted approach to sepsis treatment through their antibacterial effects, immunomodulatory capabilities, and organ-protective properties; all the machines have been sketched, as shown in Figure 3.

### 4.4. The High Safety Profile of MSCs In Vivo for Sepsis Treatment

It is of utmost importance to thoroughly assess the safety of MSCs in vivo before considering them for therapeutic use. While it is widely accepted that MSCs are not capable of tumor generation (i.e., teratoma) post-transplantation, there are potential risks [114,115]. For instance, MSCs can be captured by the original tumor in vivo, serving the tumor’s growth and potentially absorbed by the cancer cells [116,117]. This underscores the need for a comprehensive cancer biomarker screen before MSC administration, particularly for individuals (i.e., the elderly) at risk. Therefore, awareness of the potential risk of original tumor formation via physical examination is crucial. When avoiding the risk of the original tumor formation, the MSCs are safe for the patient who suffers from sepsis without tumor-associated concerns [118].

On the other hand, understanding the behavior and potential of MSCs in vivo is essential for securing their therapeutic use from the current preclinical research on animal models. The MSCs are administered intravenously and accumulate in the lung, and a very limited number of cells may migrate to the target organs, like the heart [119,120]. However, the MSCs in the lung may still improve the heart’s pumping function with ischemia via a paracrine effect. For the MSCs injected locally, like subcutaneously and into a joint, the cells may survive for weeks and exert an immune modulatory effect; Wang et al. administrated the MSCs on wound healing and found that the MSCs might accelerate the local cell regeneration and skin structure rebuilding; the host immune system will eliminate those MSCs, and there is no exogenous gene sequence detected in the organs and blood, which indicates the high safety [121]. Jiang et al. also show that the MSCs administrated in the cavity of the knee joint show positive efficacy in improving the osteoarthritis symptoms of the cynomolgus. After 9 months of monitoring, no adverse effect or tumor formation exists [13].

Therefore, MSC administration in vivo is safe due to eventual elimination by the host immune system; however, this should be satisfied when the concern of the original developing tumor is avoided via a cancer biomarker screen. For the intravenous injection of MSCs for sepsis treatment, the MSCs may respond to the stimuli of immune cytokines and activate the modulatory effect more directly and promptly with a small number of cells.

### 4.5. MSCs Having a Promising Performance for Sepsis Treatment in Preclinical Research

MSCs have been extensively studied for treating sepsis in various animal models, including mice [122,123], rats [124,125,126], and pigs [127]. Nearly all research has demonstrated significant improvements in reducing inflammation, exerting antimicrobial effects, modulating cytokines, decreasing morbidity, and protecting organ function in septic shock. Similar results have been observed in preclinical studies. In pioneering studies, Lee et al. utilized an ex vivo human lung model and induced sepsis symptoms using derivatives from *E. coli* or lipopolysaccharide (LPS), a component of the outer membrane of Gram-negative bacteria [93]. Their findings indicated that MSCs could restore alveolar fluid clearance (AFC), reduce inflammation, and exhibit antimicrobial effects partially through the secretion of keratinocyte growth factor [74,75,93].

Additionally, a preclinical study conducted in Russia administered MSCs to sepsis patients and reported an improvement in the 28-day survival rate among 30 patients. However, no statistically significant benefit was compared to the control group at day 90. This study is registered in ClinicalTrials.gov under the identifier NCT01849237. Two smaller studies involving 9 and 10 patients demonstrated symptom relief following MSC administration. They showed that human subjects could tolerate doses of up to 3 million cells per kilogram via intravenous injection in septic shock patients [76,77]. Despite these promising findings, these preclinical studies are limited by small sample sizes and require further validation in larger, controlled trials.

## 5. Limitations of MSC for Sepsis Treatment

### 5.1. Variety of Cell Quality of MSCs from the Origins

The quality and variability of MSCs have been a topic of great interest in recent research. Various studies have highlighted the significant variability in MSC concentration, total protein concentration, and cytokine profile between different sources of MSCs and among other patients [128,129]. Notably, the study by Brozovich et al. demonstrated the wide variability in MSC characteristics obtained via bone marrow aspirate concentrate (BMAC) compared with the traditional bone marrow aspiration technique [128]. This highlights the need for standardized protocols and quality assessment methods for clinical grade MSCs.

Additionally, the standardization of protocols for the derivation and characterization of human adipose tissue-derived MSCs has been addressed in the study by Debnath and Chelluri et al. [130]. This study employed a closed system protocol for clinical-grade stem cell derivation, emphasizing the importance of detailed characterization of MSCs for tissue regeneration and repair. Moreover, the efficacy of autologous bone marrow MSCs in knee cartilage repair has been studied extensively, revealing clinically relevant improvements in pain, function, and histological regeneration [131,132]. However, methodological defects in some studies have been identified, emphasizing the need for standardized guidelines and quality assessment methods for MSC-based therapies.

On the other hand, the source of serum has been identified as a critical determinant for the in vitro replicative senescence of human bone marrow-derived MSCs, affecting cell cycle regulation and the levels of heat shock proteins [133]. The findings above underscore the importance of carefully selecting the serum source in MSC culture protocols to ensure the maintenance of cell quality. However, several significant challenges remain in the clinical development of MSCs as a potential treatment for septic shock, particularly in both clinical and cell processing/manufacturing aspects. Specifically, these challenges include determining the appropriate MSC dose, choosing between fresh and cryopreserved MSC products, and identifying the most relevant clinical outcomes to evaluate. Furthermore, given that MSC therapy is highly personalized, there is no standardized protocol for critical factors such as dosing, treatment duration, or clinical endpoints. Most preclinical use of MSCs heavily relies on the physician’s evaluation and judgment [118].

Overall, the literature on MSCs underscores the importance of standardization, quality assessment, and careful consideration of factors such as cell source, culture conditions, and application in clinical therapy. These considerations are vital for advancing the therapeutic potential of MSCs in regenerative medicine and tissue engineering.

### 5.2. MSCs Are Administrated In Vivo and Are Easily Affected by Drugs

One crucial aspect that affects the quality of MSCs is their response to drugs. Several studies have explored the potential of MSCs in therapy for different conditions. For instance, Wang et al. summarized the mechanism of intestinal fibrosis and discussed the prospects of MSC therapy for this condition [134]. Their work sheds light on the potential implications of drugs on the quality of MSCs, especially in intestinal fibrosis. Similarly, a study by Zhao et al. outlined the development and investigational new drug application of MSC-based therapeutic products, providing insights into the regulatory aspects and quality management, which are crucial for understanding how drugs may affect the quality of MSCs [135]. Furthermore, Mariñas-Pardo et al. demonstrated the in vivo efficacy of allogeneic adipose-derived MSCs for treating osteoarthritis-associated lameness in horses, highlighting the importance of quality control and safety of MSC-based treatments when impacted by pharmacological factors [136].

Additionally, the review by Wang et al. summarized the research progress of MSCs from various sources in repairing articular cartilage injury, emphasizing the importance of understanding how different drugs may affect the source and quality of MSCs [137]. These findings provide valuable insights into understanding how pharmacological interventions influence the quality of MSCs. Moreover, a study by Shao et al. explored the effects of glycolysis on the metabolism of osteoblasts, which has implications for the quality and function of MSCs, especially under the influence of pharmacological agents that affect cellular metabolism [138].

### 5.3. Safety Concern of MSCs on Pre-Tumor Growth

Although the MSCs have low immunity and are safe for ordinary administration in most cases like autoimmune disease and degraded disease. Moreover, unlike the iPSC, which may form the teratoma in vivo, there is no tumor formation risk of MSCs themselves post-transplantation; usually, those administrated MSCs may be eliminated by the host immune system from several weeks to months. However, their interactions with pre-existing tumors or pre-tumor conditions raise questions about safety due to their capacity to influence the tumor microenvironment. Klopp et al. reviewed that MSCs exhibit tumor-homing properties, which can cause them to localize to pre-tumor or tumor environments, potentially supporting tumor development. [139] Spath et al. also reported that MSCs can promote tumor growth by secreting factors like VEGF, IL-6, and TGF-β, which enhance angiogenesis and create a supportive microenvironment for tumor cells [140].

Moreover, MSC’s immune modulatory ability can suppress immune responses and limit the body’s natural anti-tumor immunity, allowing pre-tumor cells to evade immune detection and grow [141].

In some cases, MSCs can interact with cancer stem cells, potentially promoting their capacity to sustain tumor growth and resist therapeutic interventions, including the induction of chemotherapy resistance through the release of platinum-induced fatty acids [142]. Therefore, careful evaluation of the tumorigenic risk of MSCs, particularly in clinical settings, is critical. Those evaluations can be achieved by multiple methods, including tumor biomarker screening, computed tomography (CT) scan, magnetic Resonance Imaging (MRI), and positron Emission Tomography Scan (PET).

Overall, MSCs are widely researched for their therapeutic potential, but their safety, particularly regarding tumor growth, is an area of concern. These studies mentioned above contribute to our understanding of how tumors can impact MSCs and highlight the importance of considering safety factors in the context of MSC-based therapies. Further research in this area is essential to comprehensively assess the interaction between tumors and the administration of MSCs, providing important insights for developing effective and safe MSC-based treatments.

## 6. The Avenue of Increasing MSC Function for Sepsis Treatment

### 6.1. MSC Priming Enhances Its Capability of Immunomodulation and Trophism

To enhance MSC’s therapeutic utility, various priming strategies have been explored [82,143]. Pierce and Kurata demonstrated that priming with toll-like receptor three agonist poly(I:C) can enhance the content of innate immune defense proteins in extracellular vesicles derived from MSCs, suggesting a potential for improved therapeutic outcomes [144]. Moreover, Noronha et al. revealed that growth factor priming of murine MSCs critically determines their functionality, emphasizing the impact of culture conditions on the fate of MSCs and their translational relevance [145].

Similarly, Hezam et al. highlighted the superior protective effects of PGE2 priming of MSCs against acute lung injury, indicating the potential of priming to enhance the therapeutic efficacy of MSCs [146]. Yasan and Gunel-Ozcan discussed the impact of hypoxia and hypoxia-mimetic agents as potential priming approaches to empower MSCs, shedding light on the effects of various physical hypoxic conditions and hypoxia-mimetic agents on MSCs at cellular and molecular level [147]. In a different context, Shin et al. demonstrated that priming MSCs with α-synuclein enhances their neuroprotective properties, suggesting the potential of MSC priming for neurological disorders [148]. Zolfaghari et al. explored the effect of poly I:C or LPS priming on the therapeutic efficacy of MSCs in an adjuvant-induced arthritis rat model, highlighting the potential of priming techniques in improving the therapeutic effects of MSCs in arthritic disorders [149].

Furthermore, the literature presents other priming strategies such as high-resolution surface acoustic wave contactless patterning [150], combination with gonadotropin therapy [151], enriched environment [152], betulinic acid treatment [153], pro-inflammatory cytokine priming [154], and 3D culture with interferon-γ priming [155]. These studies demonstrate the diverse approaches and potential implications of priming MSCs to enhance their therapeutic capabilities for various clinical applications. The review by Miceli et al. further consolidates the understanding that different priming strategies can improve the distinct therapeutic capabilities of MSCs, providing potential implications for their clinical use [156]. In conclusion, this literature review illustrates the current landscape of research on MSC priming to enhance their therapeutic utility, and it underscores the importance of considering various priming strategies in developing effective MSC-based therapies.

### 6.2. Exosomes Derived from MSCs May Achieve Non-Cell Treatment for Sepsis

MSCs have long been recognized as a promising tool for cell-based therapy due to their regenerative potential [157,158]. However, emerging evidence suggests that the therapeutic effects of MSCs could be attributed to the exosomes they secrete rather than their direct differentiation and engraftment [159]. These exosomes, small membrane-bound vesicles, are rich in bioactive molecules such as proteins and microRNAs and have been shown to mediate various beneficial effects in different disease contexts [160,161].

Recent studies have shed light on the potential of MSC-secreted exosomes as a novel cell-free-based therapy for various chronic respiratory diseases, including chronic obstructive pulmonary disease (COPD), asthma, pulmonary fibrosis, and pulmonary arterial hypertension [162]. This finding opens up new avenues for developing exosome-based therapies that could overcome the limitations (e.g., live/dead test, freeze–thaw cycle, and daily maintenance) associated with cell-based approaches, such as potential tumorigenicity and immunogenicity [163]. Moreover, the pro-angiogenic potential of exosomes derived from oral leukoplakia and carcinoma-associated MSCs has been linked to high matrix metalloproteinase 1 (MMP1) content, providing valuable insights for intervention strategies targeting the secretion of MSC-derived exosomes to impede carcinogenesis [164].

In line with these findings, it has been proposed that the functional mechanism of MSC exosomes is predominantly protein-based rather than RNA-based, expanding our understanding of their biological relevance and therapeutic potential [159]. This knowledge is crucial for developing exosome-based therapies, as it emphasizes the importance of considering the physical presence of exosomes and their specific biochemical functionality and ability to elicit timely biochemical responses. Furthermore, MSC-derived exosomes have demonstrated the ability to regulate immune responses and inflammation in various disease models, including dextran sulfate sodium (DSS)-induced acute colitis and pressure overload-induced heart failure, highlighting their broad therapeutic potential [160,165].

Identifying specific regulatory factors, such as let-7c shuttled by adipose-derived MSC exosomes to modulate macrophage polarization, further exemplifies the intricate molecular mechanisms through which MSC exosomes exert their therapeutic effects [165]. These insights pave the way for developing tailored exosome-based therapeutics for various conditions, including skin inflammation and barrier dysfunction [166,167]. In addition, exosomes have also been instrumental in diagnostic applications, with the potential for detecting low-abundant mutant copies of epidermal growth factor receptor (EGFR) in non-small cell lung cancer (NSCLC) using combined exosomal nucleic acid analysis in plasma and pleural fluid [168]. These findings collectively highlight the burgeoning interest in MSC-derived exosomes as a promising avenue for non-cell therapy, offering a wealth of possibilities for developing novel therapeutics and diagnostic tools for various diseases.

### 6.3. Genetic Engineering on MSCs May Enhance Its Performance in Applications

Genetic modification has emerged as a promising approach to enhance the therapeutic capacity of MSCs for cell therapy [169]. A study by Thakor et al. further supports the potential of genetic engineering in MSCs, demonstrating that serum-compatible pullulan–spermine/DNA lipoplexes can effectively modify rat MSCs, providing a straightforward technology for genetic engineering under physiological niche-like conditions [170]. Ebrahim et al. delve into the effects of genetic modification of MSCs for neurological disease therapy, focusing on the impact on phenotype, cell behavior, proliferation, and differentiation ability of these cells [171].

Meanwhile, the study by Ball et al. addresses the alteration of transgene expression in bone marrow-derived MSCs after cryopreservation, presenting an “off-the-shelf” option for fracture repair [172]. Another intriguing application of genetic modification is highlighted in Margiana’s review, which summarizes the potential of MSC transplantation for enhancing spermatogenesis in non-obstructive azoospermia [173]. Moreover, the study by Ebrahim et al. demonstrates the restoration of postnatal oogenesis in chemo-ablated ovaries through the genetic engineering of MSCs, shedding light on the promising impact of MSC-based therapy [151].

However, in the context of therapeutic efficacy, Zhang et al. reveal the adverse effects of excessive branched-chain amino acid accumulation on the retention and cardioprotection of intramyocardially injected MSCs, emphasizing the need for careful consideration of metabolic factors in genetic modification strategies [174]. In regenerative medicine, Damasceno et al. offer valuable insights into the different genetically engineered tools employed for MSC modification, emphasizing the factors investigated in multiple fields where genetically engineered MSCs have been tested [175]. Meanwhile, Cheng et al. explore the engineering of MSC-derived exosomes as a promising cell-free therapy for osteoarthritis, introducing novel approaches for advanced MSC-based therapies [176]. In conclusion, genetic modification holds immense potential in enhancing the therapeutic capacity of MSCs for cell therapy, although with some safety, but recent studies showcasing its diverse applications across different disease conditions and regenerative medicine scenarios.

## 7. Conclusion and Perspectives

The typical use of antibiotics is a significant factor in the rising issue of antibiotic resistance, which makes the management of sepsis very challenging. As antibiotic treatments lose their effectiveness because of resistant agents, patients spend more days in the healthcare system, and the cost of care significantly increases. The more the pathogens become resistant, the fewer effective antibiotics are available, and patient options become limited with time. The surge of resistance threatens to push the world into a pre-antibiotic era, where even minor infections may bring death from uncontrolled bacteria. The pressure on the health system, individual health, and the need for effective treatment is substantial now and in the future.

Using MSCs in sepsis therapy epitomizes significant progress in medical science. The efficacy of this therapy is rooted in the unique immunological characteristics of MSCs, which can arrest immune reactions to ensure the killing of the bacteria with AMPs while preventing overactivation, which can trigger septic shocks. In addition, the cells have a limited lifespan of only a few weeks in vivo, reducing the risk of severe adverse effects following prolonged treatment [121]. The use of MSCs in sepsis therapy has expanded over the last two decades to other critical conditions, notably COVID-19, where it has demonstrated the capacity to control the cytokine storm, a deadly hyperimmune response [177]. The broad applicability and remarkable safety profile of MSC therapy underpin the remedy’s potential as a revolutionary treatment choice for sepsis and many other inflammatory diseases.

While MSCs’ antibacterial, anti-inflammatory, and tissue-producing qualities make them a highly promising avenue in sepsis treatment (Figure 3), the novelty of their mechanism of action warrants further investigation. The role of MSCs in treating sepsis is new and could either serve as an alternative personalized avenue to modern resistance-compromise antibiotics or alleviate their reliance. MSCs still need to be ready to substitute for antibiotics since direct antimicrobial action is still the most critical aspect of controlling infections in the first place.

The role of MSC therapy, however, would be suitable as an adjunct intervention for sepsis patients that would improve patients’ recovery time by suppressing hyperinflammatory responses, protecting organ function, and producing tissue while possibly supporting high doses or long-term exposure to antibiotics. This approach not only addresses the most severe present danger, which is a bacterial disease but also contributes to the long-term future goal of antibiotic preservation. As MSC research shows, the nuanced mechanism of action associated with the immune system and the advanced healing potential of MSCs could be a critical factor in a more worthwhile and sustainable future against sepsis and related infections.

## Figures and Tables

**Table 1:** Novel biomarker candidates for early stage of sepsis diagnostics.

Biomarkers	Category	Function	Ref.
PAMPs	Innate response	Microbial motifs recognized by host cell pattern-recognition receptors	[45]
DAMPs	Innate response	Molecular alerts of host system damage	[46]
Calprotectin	Innate response	Released from neutrophils in response to bacterial infections, increasing within hours	[47,48]
MCP1	Chemokine	Endothelial cells and monocytes secrete MCP-1 (i.e., C-C motif chemokine ligand (2) to trigger inflammation.	[49]
PTX-3	Cytokine	Triggers early inflammation by activating the classical complement pathway and aiding pathogen recognition by macrophages and dendritic cells.	[50,51]
sTNFR	Cytokine	Early pro-inflammatory cytokines were studied to assess their correlation with sepsis mortality, typically linked to an exaggerated innate immune response	[52]

Abbreviations: PAMPs: Pathogen-associated molecular patterns; DAMPs: Damage-associated molecular patterns; MCP1: Monocyte Chemoattractant Protein 1; PTX 3: Pentraxin; sTNFR: soluble molecules of tumor necrosis factor receptors.

**Table 2:** Mesenchymal stem cells (MSCs) that have been administrated for partial human diseases, including the preclinical effects of MSC therapy for sepsis.

MSC Type	Disease	Year	Patient No.	Conclusion
ADSC	Acute ischemic stroke	2014 [57]	10	Potential time window for the intravenous administration of allogeneic ADSC and improved efficacy when performed within 2 weeks after stroke.
BM-MSC	Autism spectrum disorders	2020 [58]	254	After the transplantation, the change in score: ISAA was positive (94.48% of patients); CARS improved (95.27% of patients); brain activity improved (86/86 in FDG-PET CT)
UC-MSC	Cerebral palsy	2020 [59]	19	The activities of daily living, comprehensive functional assessment, and Gross motor function measurement—88 scores were significantly improved compared to pretransplant and control groups.
BM-MSCs	Chronic stroke	2019 [60]	36	Based on serial examination, electrogram, laboratory, and computed tomography scans of chest/abdomen/pelvis, the therapy was safe and well-tolerated.
BM-MSCs	Spinal cord injury	2021 [61]	41	There was a notable enhancement in the ASIA total score, pinprick score, and light touch, as well as the IANR-SCIFRS total score and sphincter score following transplantation when compared to pre-transplantation assessments.
BM-MSCs	Multiple sclerosis	2017 [62]	10	There has been a general trend of enhancement observed in the Expanded Disability Status Scale and secondary clinical tests, which encompassed mobility, cognitive function (Mini-Mental Status Examination), and ophthalmological assessments.
BM-MSCs	Acute respiratory distress syndrome	2019 [63]	60	Elevate the acute physiology and chronic health evaluation III score, enhance minute ventilation, and achieve a reduction in inflammation.
UC-MSC	Bronchopulmonary dysplasia	2021 [64]	33	Transplantation of MSCs markedly enhanced the condition of patients with severe Bronchopulmonary Dysplasia.
UC-MSC	COPD	2020 [65]	20	All patients experienced enhancements in their Modified Medical Research Council scores and COPD Assessment Test outcomes.
UC-MSC and ADSC	COVID-19	2021 [66]	210	Decrease in inflammatory responses and enhancement in survival rates.
UC-MSC	COVID-19	2021 [67]	24	UC-MSC infusions have demonstrated safety and may offer therapeutic benefits for individuals with ARDS in the context of COVID-19.
UC-MSC	COVID-19	2021 [68]	24	Recipients of UC-MSCs exhibit a marked increase in plasma sTNFR2 levels, along with a significant reduction in tumor necrosis factor α and β levels, when compared to control subjects.
BM-MSC	Idiopathic pulmonary fibrosis	2019 [69]	10	Enhanced performance in carbon monoxide diffusing capacity, six-minute walk distance, and increased forced vital capacity.
ADSC	Type 1 diabetes	2021 [70]	7	Marked enhancement in basal C-peptide levels and HbA1C measurements following transplantation relative to pre-transplantation values.
BM-MSCs	Type 2 diabetes	2021 [71]	25	There was a minor decrease in HbA1c levels within the initial three months post-administration; however, the levels normalized after six months and subsequently rose.
BM-MSCs	Skin wound	2017 [72]	40	BM-MSC group exhibited a notably higher healing rate compared to the group receiving traditional treatment in terms of percentage of burn coverage and duration of hospital stay.
ADSC	Refractory angina	2019 [73]	41	Enhanced cardiac symptoms were observed, yet there was no improvement in exercise capacity.
BM-MSC	Sepsis	2009 [74]	NA	Restoration of alveolar fluid clearance via sodium-dependent transport mechanisms (LPS induced on ex vivo human lung model).
BM-MSC	Sepsis	2013 [75]	NA	Restore alveolar fluid clearance (AFC), reduce inflammation, and demonstrate antimicrobial effects, partly through the secretion of keratinocyte growth factor (*E. coli* induced on ex vivo human lung model).
MSCs (undefined)	Sepsis	2013	30	Improved the survival rate of 28-day period. However, its beneficial effect shows no significancy to control on day 90. ClinicalTrials.gov: NCT01849237.
BM-MSC	Sepsis	2018 [76]	9	The infusion of allogenic BM-MSCs, up to 3 million cells/kg, appears safe in septic shock patients. ClinicalTrials.gov: NCT02421484.
ADSC	Sepsis	2022 [77]	10	Improved early survival rates in sepsis for 10 patients, but larger randomized controlled studies are needed. ClinicalTrials.gov: NCT05283317.

Abbreviations: ADSC stands for adipose-derived stem cells, BM-MSC for bone marrow-derived mesenchymal stem cells, and UC-MSC for umbilical cord-derived mesenchymal stem cells. COPD stands for Chronic Obstructive Pulmonary Dysplasia. NA stands for Not Available. ARDS stands for Acute Respiratory Distress Syndrome.

**Table 3:** Mesenchymal stem cell (MSC) therapies have been approved by regulatory agencies worldwide.

Cell Type	Brand	Indication	Company	Approved Area	Approved Date
Allogeneic Bone Marrow-Derived Mesenchymal Stem Cells	Alofisel	Crohn’s Disease with Anal Fistula	TiGenix NV	Europa	2018
Allogeneic Bone Marrow-Derived Mesenchymal Stem Cells	Alofisel	Crohn’s Disease with Anal Fistula	TiGenix NV	Japan	2021
Autologous Adipose-Derived Mesenchymal Stem Cells	Guepistem	Crohn’s Disease with Anal Fistula	Anterogen	Korea	2012
Autologous Bone Marrow-Derived Mesenchymal Stem Cells	Hearticellgram-AMI	Acute Myocardial Infarction	FCB-Pharmicell	Korea	2011
Autologous Limbal Stem Cells	Holoclar	Burn-induced Limbal Stem Cell Deficiency	Chiesi Farmaceutici	Europa	2015
Autologous Mesenchymal Progenitor Cells	MPC	Damaged Bone Tissue Repair	Mesoblast	Australia	2010
Bone Marrow-Derived Mesenchymal Stem Cells	MultiStem	Ischemic stroke	America Stem Cell	United States	2012
Bone Marrow-Derived Mesenchymal Stem Cells	Prochymal	Graft-Versus-Host Disease	Osiris Therapeutics	United States	2005
Bone Marrow-Derived Mesenchymal Stem Cells	Prochymal	Crohn’s Disease	Osiris Therapeutics	United States	2009
Bone Marrow-Derived Mesenchymal Stem Cells	Prochymal	Type 1 Diabetes	0siris Therapeutics	United States	2010
Bone Marrow-Derived Mesenchymal Stem Cells	Ryoncil (Prochymal)	Graft-Versus-Host Disease	Osiris Therapeutics	Canada	2012
Bone Marrow-Derived Mesenchymal Stem Cells	Stempeucel	Buerger’s disease	Stempeutics	India	2020
Bone Marrow-Derived Mesenchymal Stem Cells	Temcell	Graft-Versus-Host Disease	Mesoblast	Japan	2016
IPSC-Derived Mesenchymal Stem Cells	Cymerus	Graft-Versus-Host Disease	Cyanta Therapeutics	United States	2018

## Data Availability

Data are available per the readers’ request to the authors.
